# *In situ* MgO nanoparticle-doped Janus electrospun dressing against bacterial invasion and immune imbalance for irregular wound healing

**DOI:** 10.1093/rb/rbae107

**Published:** 2024-08-23

**Authors:** Tao Zhou, Yedan Chen, Liangmin Fu, Shan Wang, Haihu Ding, Qiaosheng Bai, Jingjing Guan, Yingji Mao

**Affiliations:** Department of Orthopedics, The First Affiliated Hospital of Bengbu Medical University, Bengbu, 233004, China; Anhui Province Key Laboratory of Tissue Transplantation, Bengbu Medical University, Bengbu, 233030, China; School of Life Sciences, Bengbu Medical University, Bengbu, 233030, China; Department of Plastic Surgery, The First Affiliated Hospital of Bengbu Medical University, Bengbu, 233004, China; Anhui Province Key Laboratory of Tissue Transplantation, Bengbu Medical University, Bengbu, 233030, China; Department of Plastic Surgery, The First Affiliated Hospital of Bengbu Medical University, Bengbu, 233004, China; School of Life Sciences, Bengbu Medical University, Bengbu, 233030, China; Anhui Nerve Regeneration Technology and Medical New Materials Engineering Research Center, Bengbu Medical University, Bengbu, 233030, China; Anhui Province Key Laboratory of Tissue Transplantation, Bengbu Medical University, Bengbu, 233030, China; Anhui Province Key Laboratory of Tissue Transplantation, Bengbu Medical University, Bengbu, 233030, China; Department of Orthopedics, The First Affiliated Hospital of Bengbu Medical University, Bengbu, 233004, China; Anhui Province Key Laboratory of Tissue Transplantation, Bengbu Medical University, Bengbu, 233030, China; Department of Orthopedics, The First Affiliated Hospital of Bengbu Medical University, Bengbu, 233004, China; School of Life Sciences, Bengbu Medical University, Bengbu, 233030, China; Department of Plastic Surgery, The First Affiliated Hospital of Bengbu Medical University, Bengbu, 233004, China; Anhui Nerve Regeneration Technology and Medical New Materials Engineering Research Center, Bengbu Medical University, Bengbu, 233030, China

**Keywords:** *in situ* electrospinning, Janus structure, immunomodulation, antimicrobial, wound healing

## Abstract

Owing to the unpredictable size of wounds and irregular edges formed by trauma, nanofibers’ highly customizable and adherent *in situ* deposition can contribute to intervention in the healing process. However, electrospinning is limited by the constraints of conventional polymeric materials despite its potential for anti-inflammatory and antimicrobial properties. Here, inspired by the Janus structure and biochemistry of nanometal ions, we developed an *in situ* sprayed electrospinning method to overcome bacterial infections and immune imbalances during wound healing. The bilayer fiber scaffold has a hydrophobic outer layer composed of polycaprolactone (PCL) and a hydrophilic inner layer composed of gelatin, poly(L-lactic acid) (PLLA), and magnesium oxide nanoparticles, constituting the PCL/PLLA-gelatin-MgO (PPGM) electrospun scaffold. This electrospun scaffold blocked the colonization and growth of bacteria and remained stable on the wound for continuous anti-inflammatory properties to promote wound healing. Furthermore, PPGM electrospinning modulated collagen deposition and the inflammatory microenvironment in the full-thickness skin model, significantly accelerating vascularization and epithelialization progression. This personalized Janus electrospun scaffold has excellent potential as a new type of wound dressing for first aid and wound healthcare.

## Graphical abstract

**Figure:**
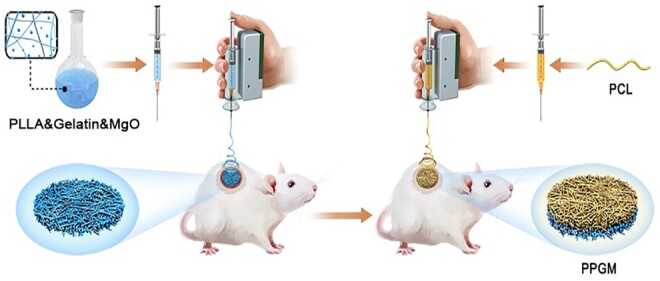

## Introduction

The skin is the largest organ in the body and is the first barrier to invasion by exogenous substances [1]. Wound healing undergoes periods of hemostasis, inflammation, hyperplasia and remodeling and may be accompanied by various internal and external factors that impede wound healing [2]. High-quality repair of full-thickness skin wounds in harsh environments is a widespread concern. Acute trauma is often associated with irregular wound sizes and margins. Therefore, it is crucial to formulate a surface dressing capable of accommodating uneven injuries, effectively closing wounds, resisting external contamination and maintaining optimal gas exchange. The selection of wound dressings has shifted from traditional gauze and cotton to advanced polymer compounds, functionalized hydrogels and micro/nanofibers. The active ingredient is delivered to the wound via diffusion by encapsulating the drug in these matrices. Although progress has been made in previous studies, further exploration is required to address the challenges of dressing for irregular wounds.

The wound is susceptible to infection by pathogenic microorganisms during healing, which can impede recovery. Antibiotics are usually loaded within wound dressings to achieve the desired antimicrobial effect; however, this can lead to the overuse of antibiotics and trigger side effects [3]. Therefore, metal nanoparticles, such as silver [4], copper [5], zinc [6], magnesium [7] and their derivatives, are gaining attention for their antimicrobial properties in treating infected wounds owing to the gradual clarification of their physicochemical properties. This approach offers a new way to combat antibiotic misuse and eliminate bacteria. However, concerns have been raised regarding its biotoxicity and potential implications for the body. Meanwhile, various metallic elements in the human body have become a topic of significant interest with advances in autologous research. Magnesium ions are abundant in the body and are essential in maintaining microenvironmental homeostasis, cell proliferation and differentiation [8]. Magnesium oxide nanoparticles (MgO NPs) kill bacteria via the non-reactive oxygen species (ROS)-mediated pathway, which may originate from the disruption of the bacterial cell membrane [9] and the alteration of bacterial survival by magnesium ions through the ribosome [10]. Meanwhile, magnesium may attenuate inflammation by altering the macrophage phenotype [11] and modulating inflammatory factor release within the NF-κB pathway [12]. Therefore, magnesium and its compounds have been selected as a new generation of antibacterial and anti-inflammatory agents for application in novel delivery technologies, such as hydrogels [13], microspheres [14] and electrospinning [15].

Electrospinning is a simple and efficient method for preparing micro/nanofibers. These nanofibers possess different physical properties resulting from different preparation processes. Still, they all exhibit high specific surface areas and porosities [16], which are of great interest in the fields of the environment, smart wearables, and biomedicine. The nanofibrous scaffold provides a micro/nanoscale artificial extracellular microenvironment for cell adhesion and growth. In complex environments such as traffic accidents and battlefield trauma, the objective is to manage wounds in a simple, rapid and infection-free manner. The customizability and high adherence of *in situ* electrospinning make it suitable and applicable in first aid compared to the common externally prepared and secondary processed conventional wound dressings. Because conventional dressings must be transferred to the defect, this may cause an imperfect correspondence with the wound and potentially infected cavities [17]. For instance, the timing and scope of spraying can be individualized to suit site conditions and various wound geometries, significantly reducing wound treatment time [18]. Asymmetrically wetted electrospinning is an emerging approach for exerting capabilities in addition to the purposeful role of the additional components.

Asymmetric wetting electrospinning involves the application of a Janus structure and typically involves two sides with different hydrophilicities opposite each other. The hydrophobic outer layer is often sprayed with polycaprolactone (PCL) [19, 20], poly(lactide-co-glycolide) (PLGA) polymers [21] and pretreated using octadecyl trichlorosilane [22] to reduce bacterial adhesion and exogenous pathogen invasion to achieve a barrier effect. The sealing ability of the membrane is enhanced by its hydrophobicity, which prevents reverse liquid loss, particularly during sustained blood loss or tissue fluid evaporation. The hydrophilic inner layer is often composed of gelatin [23], silk protein [24] and chitosan [25], which absorb exudates and release the loaded ingredients. Among the available options, gelatin has attracted attention owing to its wide range of biological sources and compatibility. However, hydrophilic materials often lack the mechanical properties necessary to satisfy practical requirements. Therefore, poly(L-lactic acid) (PLLA) is used to provide the required mechanical support [26]. The mechanical properties and excellent biocompatibility of PLLA are comparable with that of PLA while crucially changing from its original hydrophobic state to a hydrophilic state. The design of this distinctive structure has inspired innovative wound dressings.

**Figure 1.**
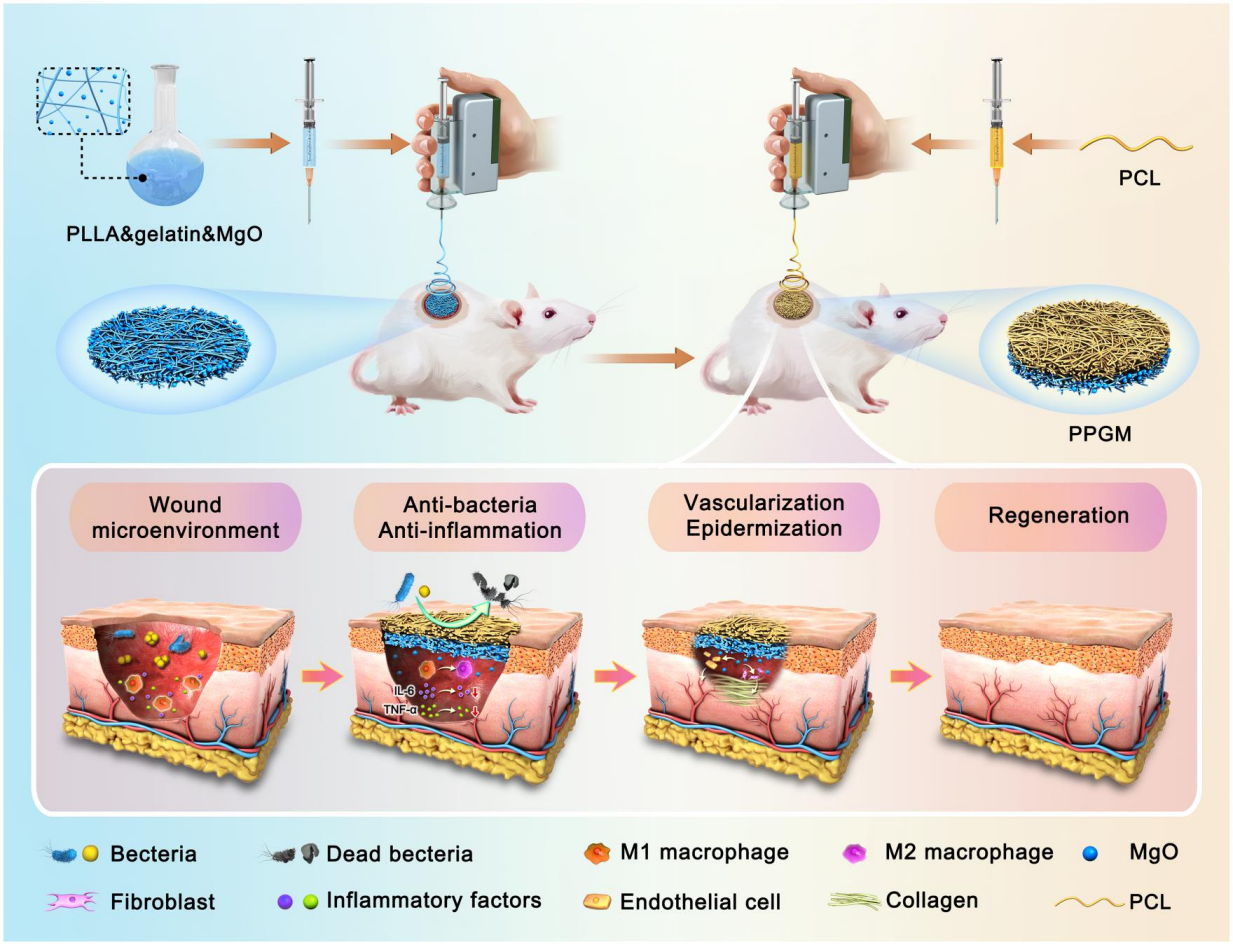
Schematic illustration of the fabricated electrospinning scaffold inspired by Janus structures and the effect of vascularization and epithelialization to accelerate wound healing (PPGM, PCL/PLLA-Gelatin-MgO electrospun scaffold).

Herein, we fabricated a PCL/PLLA-Gelatin-MgO (PPGM) electrospun nanofibrous scaffold with high adhesion, barrier, antibacterial and anti-inflammatory properties using an *in situ* electrospinning technique inspired by the Janus structure and metal ion biochemistry (Figure 1). Homogenized PLLA/Gelatin/MgO and PCL solutions were rapidly sprayed into the wound by electrospinning using a handheld electrospinning device. By targeting the wound microenvironment, the PPGM electrospun scaffold acted externally as a barrier and internally with its antimicrobial and anti-inflammatory properties, promoting vascularization and epithelialization of the wound and accelerating wound healing. Comprehensive *in vitro* and *in vivo* evaluations demonstrated that PPGM electrospinning exerted diverse internal and external functions, offering a rapid, convenient and efficient option for first aid and wound healthcare in the future.

## Materials and methods

### Materials

Polycaprolactone (EFL-PCL-80k) and gelatin (EFL-GEL-001) were purchased from EFL-Tech Co. (Suzhou, China). PLLA was purchased from Jinandaigang Biochemical Co. (Jinan, China). Dichloromethane (DCM) and *N*,*N*-Dimethylformamide (DMF) were obtained from Shanghai Macklin Biochemical Co. (Shanghai, China). 1,1,1,3,3,3-Hexafluoro-2-propanol (HFIP) was purchased from Shanghai Aladdin Co. (Shanghai, China). As previously reported, MgO NPs were synthesized [27].

### Preparation of the PPGM electrospinning

The PPGM electrospinning solution was prepared according to a previous study [28]. PPGM electrospinning was synthesized via the spray using PCL, PLLA, gelatin and MgO NPs. The outer hydrophobic electrospinning solution consisted of a mixture of PCL (1.5 g) dissolved in 7 ml DCM and 3 ml DMF. Meanwhile, the inner hydrophilic electrospinning solution was prepared by dissolving 0.56 g PLLA and 0.24 g gelatin in 10 ml HFIP to make an 8% wt mixture. Based on 10 ml of the HFIP solution, 0, 25, 50 and 100 mg of MgO NPs were added, named PPG, PPGM-L, PPGM-M and PPGM-H, respectively. The two solutions were magnetically stirred for 30 min at 20°C to obtain a homogeneous and stable solution.

The solution was transferred into a 5 ml syringe with a 22G metal needle and placed on an HHE-1 handheld electrospinning device (JUNADA, China). Round coverslips were used for electrospinning (working model: 10 kV voltage, 10 cm collecting distance, 90° collecting area). The inner hydrophilic membrane was initially prepared, followed by the direct application of the external hydrophobic membrane through a spray process.

### Characterization of PPGM electrospinning

The surfaces and chemical compositions of the PPGM electrospun samples were analyzed by scanning electron microscopy (SEM, TESCAN MIRA LMS, Czech Republic). Transmission electron microscopy (TEM, JEOL JEM-F200, Japan) was used to demonstrate the loading of MgO onto the electrospun membranes, which were collected on a carbon-coated copper grid.

Fourier transform infrared spectroscopy (FTIR) and X-ray diffraction (XRD) were utilized to characterize the MgO NPs and PPGM electrospun samples. A Thermo Fisher Scientific iN10 (Thermo, USA) infrared microscope was used for FTIR spectrometry analysis in the 4000–600 cm^−1^ wavelength range. SmartLab SE (Rigaku, Japan) was used for XRD analysis, detecting the samples at 2°/min between 10° and 60°. The water contact angle (WCA) contact angle measuring instrument was measured with an SDC 350KS to 10 μl deionized water droplets. The osmosis and reverse osmosis of 10 μl red ink drops on the sample surface were recorded by a digital camera (Canon, Japan).

Thermogravimetric (TG, Rigaku, Japan) analysis of PPGM electrospinning was performed at a scan rate of 20°C min^−1^ from 20°C to 800°C under an inert atmosphere. The ion concentration determined the release of Mg ions from PPGM electrospinning. Solutions from the incubated samples were collected at 37°C and 200 rpm at predetermined point intervals. An inductively coupled plasma optical emission spectrometer (ICP-OES; Agilent 5110, USA) measured Mg ion concentrations in the liquids.

### *In vitro* experiments

#### Cell culture

Human umbilical vein endothelial cells (HUVECs) and NIH/3T3s were purchased from Procell Life Science & Technology Co. The cells were incubated in Dulbecco’s modified Eagle’s medium (Gibco) supplemented with 10% fetal bovine serum and 1% penicillin-streptomycin solution in a controlled incubator at 37°C with 5% CO_2_. The medium was changed every 2 days. Raw264.7 cells were cultivated, as previously reported [29].

#### Biocompatibility analysis

The electrospun nanofibers were classified into five groups to determine the optimum concentration of magnesium ions. Briefly, 5 × 10^4^/ml HUVECs and NIH/3T3s were cultured in 96-well plates for 1, 3 and 5 days. Cell Counting Kit-8 (CCK-8, APE×BIO, K1018) was added, and the cells were incubated for 2 h in the dark at 37°C. The samples were analyzed, and the optical density (OD) was recorded at 450 nm.

To observe the effect of the PPGM electrospun scaffolds on cell viability, HUVECs and NIH/3T3s were seeded in 24-well plates at a density of 5 × 10^4^/ml for 1, 2 and 3 days according to the previous groups, respectively. A Calcein-AM/PI staining kit (Yeasen, China) was used, and the cells were incubated for 20 min. Samples were photographed using an environmental scanning electron microscope (QUANTA250, USA). Each group’s live and dead cells were counted and compared with the control group.

HUVECs and NIH/3T3s were grown in 24-well plates for 3 days to investigate the impact of the PPGM electrospun scaffolds on cell morphology and state. The plates were fixed with 4% paraformaldehyde for 15 min and incubated with fluorescein isothiocyanate (FITC)-Phalloidin (1:250, Solarbio) working solution for 30 min. The cell nuclei were stained with 4′,6-diamidino-2-phenylindole (DAPI) (10 μg/ml, Biosharp) for 10 min.

#### Scratch migration experiment

To investigate the effects of MgO NPs on cell migration capacity, different concentrations of MgO NPs (0%, 0.25%, 0.5%, 1% wt/v) were co-cultured with HUVECs or NIH/3T3s in 6-well plates and grown until 90% confluence with fresh growth medium. A 200-μl tip was used to make parallel scratches in the center of the confluent cell monolayers. Subsequently, scratch migration was observed and imaged using a fluorescence microscope at 0 and 24 h by Calcein-AM staining. Each group’s initial and final scratch areas were statistically calculated and compared with the control group using ImageJ.

#### Tube formation assay

A tube formation assay was conducted using five groups to investigate the impact of the PPGM electrospun scaffolds on the angiogenesis of HUVECs. Matrigel diluent (100 µl) was added to pre-cooled 24-well plates and solidified at 37°C for 30 min. Subsequently, 1.5 × 10^5^ HUVECs were seeded into the prepared wells and incubated for 6 h. Afterward, the cells were stained with Calcein-AM, and images were collected using a fluorescent microscope. The numbers of nodes, junctions, and meshes were counted along the length of the dot. and Tot. branching compared to the control group.

#### Immunofluorescence staining-macrophage polarization

RAW264.7 cells were stimulated and treated with the PPGM electrospinning to mimic changes in the immune microenvironment during wound repair. The distribution of M1 and M2 phenotypic macrophages was examined to investigate the effect of PPGM electrospun fiber scaffolds on macrophage polarization. RAW264.7 cells were seeded in 24-well plates at a density of 8 × 10^5^/well and incubated with a medium containing 100 ng/ml lipopolysaccharide (LPS) for 24 h at 37°C. Cells were fixed with 4% paraformaldehyde for 15 min and then sealed with 10% goat serum for 2 h. Different groups were incubated overnight 4°C with anti-CD86 (M1 phenotype marker, 1:200, Proteintech) and anti-CD206 (M2 phenotype marker, 1:200, Proteintech) primary antibodies. Subsequently, the cells were incubated with Cy3-labeled (1:1000, Proteintech) or FITC-labeled (1:1000, Proteintech) secondary antibodies for 1 h at 20°C in the dark. The cell nuclei were stained with DAPI (10 μg/ml, Biosharp). Fluorescence images were obtained under various fluorescence excitations using a fluorescence microscope (Zeiss Axio Observer Z1, Germany).

#### Antibacterial activity

*Escherichia coli* and *Staphylococcus aureus* were used as models for the inhibition loop and agar plate counting tests to evaluate antimicrobial activity. For the inhibition loop assay, a 10^6^ colony forming unit (CFU)/ml bacterial solution was homogeneously distributed on the surface of the agar medium for inoculation. The UV-irradiated samples were placed on the surface of the agar medium and gently pressed so that the samples were in complete contact with the medium. The Petri dishes were incubated at 37°C with 5% CO_2_ for 24 h. The samples were photographed using a digital camera, and the diameters of the bacteriostatic rings were measured using Vernier calipers. The bacterial coating test involved adding 1 ml of a 10^5^ CFU/ml bacterial solution to the fiber membrane and incubating for 24 h. After collecting the solution and diluting it to 10^3^ CFU/ml, 100 μl of the dilution was slowly dispersed in an LB solid medium. The plate was subsequently inverted and incubated for 24 h. Photographs were taken to record the number of colonies, and the bacterial survival rate was assessed to determine the fiber membrane inhibition rate.

$$\mathrm{Survival}\;\mathrm{rate}\;\left(\%\right)=\frac{A_{t}}{A_{0}}\;\times \;100\%$$

*A*_0_ is the number of colonies on the black control plate, and *A*_t_ is the number of colonies on the culture plates for each fiber membrane intervention.

### *In vivo* experiments

Rats were purchased from the Zhejiang Experimental Animal Center (Hangzhou, China). All procedures and treatments were approved by the Institutional Animal Care and Use Committee of Bengbu Medical College (Approval No. 2023189). Male Sprague Dawley (SD) rats (200–250 g in weight) were used to evaluate 1.5 × 1.5 cm round skin defects of the whole layer at the midline of the vertebral line. Subsequently, the electrospun membrane was sprayed on the area of full skin defects, and the group, without any intervention, was designated as the control. Wound healing was captured using a digital camera on the 3rd, 7th, and 14th day, and quantitative analysis was performed using ImageJ software. The collected skin tissues were immersed in formalin for 48 h. Paraffin-embedded skin sections were stained with hematoxylin eosin (HE) and Masson’s trichrome. Skin tissue slices were processed by immunofluorescence staining. The sections were incubated overnight at 4°C with primary antibodies against CD31 (1:100, affinity), α-SMA (1:100, affinity), CD86 (1:100, affinity), CD206 (1:100, affinity), IL-6 (1:100, affinity), TNF-α (1:100, affinity), COL I (1:100, affinity) or COL III (1:100, affinity). Subsequently, the sections were stained with Cy3-labeled (1:1000, Proteintech) or FITC-labeled (1:1000, Proteintech) secondary antibodies at room temperature for 2 h. The sections were counterstained with DAPI (5 μg/ml) for 10 min, followed by washing with PBS three times, each lasting 5 min. Wound healing was calculated using the formula:

$$\mathrm{Wound}\;\mathrm{healing}\;\left(\%\right)=100-100\times \frac{S_{0}-S_{x}}{S_{0}}$$

*S*_0_ is the wound area on day 0, and *S_x_* is the wound area on days 3, 7 and 14.

### Statistical analysis

All results were expressed as means ± standard deviation, and all data were processed using GraphPad Prism (GraphPad Software, Inc., USA). Differences were considered statistically significant at *P *<* *0.05. Statistically significant differences are expressed as not significant (ns, *P *>* *0.05); * (*P *<* *0.05), **(*P *<* *0.01) and *** (*P *<* *0.001). Results are presented as means ± standard deviation (SD).

## Results and discussion

### Characterization of PPGM electrospinning

The synthesis and application of PPGM electrospinning for wound healing are shown in Figure 1. The handheld electrostatic spinning device was used to first spray a hydrophilic membrane on the trauma surface. Subsequently, the hydrophobic membrane was deposited *in situ* on top of the hydrophilic membrane in layers. The relevant characterization of the MgO NPs was shown in Supplementary Figure S1. The particle size was around 40 nm, and energy-dispersive X-ray spectroscopy (EDS) showed an overlapping of Mg and O elements. We performed PPGM electrospinning, which depended on mixing different masses of MgO NPs (PPG, PPGM-L, PPGM-M, and PPGM-H). A smaller deposition area was achieved owing to the shorter electrospinning collection distance of the handheld device. Precision deposition enables precise customization of the dressing shape for irregular wounds (Supplementary Figure S2).

**Figure 2.**
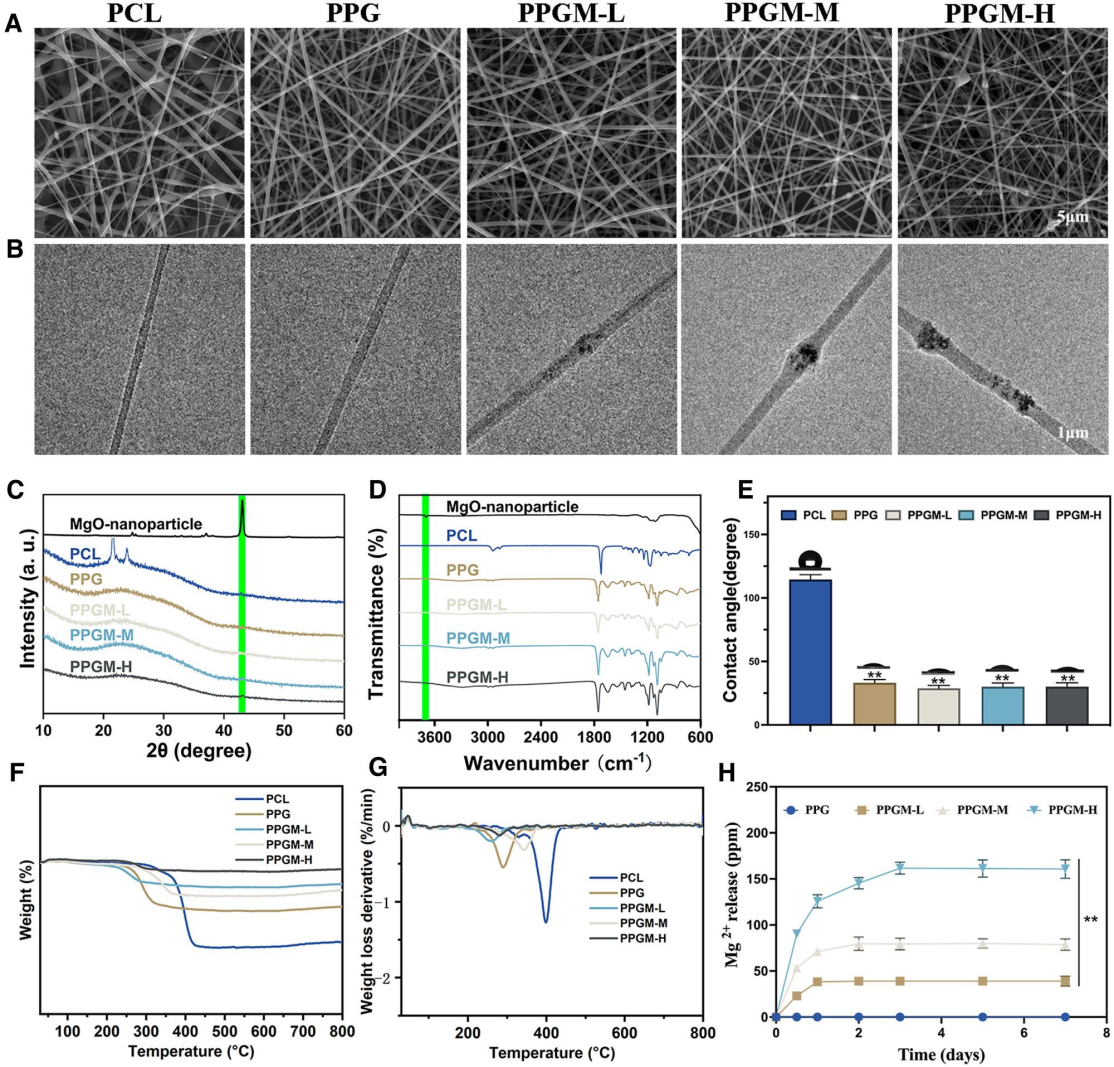
Characterization of the MgO NPs and PPGM electrospinning membranes. (**A**) Representative SEM images. (**B**) Representative TEM images. (**C**) XRD patterns. (**D**) Fourier transform infrared spectra. (**E**) WCA. (**F**, **G**) TG analysis. (**H**) *In vitro* release profile of Mg^2+^ in ultrapure water (*n* = 3, **P <* *0.01).

The morphological properties of PCL, PPG, PPGM-L, PPGM-M and PPGM-H electrospun samples were analyzed by SEM (Figure 2A). The electrospun fibers were uniformly thick; however, their distribution was disordered. The scaffolds had a well-defined three-dimensional structure owing to the composite bilayer membrane and the stacking of nanofibers. The elemental compositions of the electrospun samples were analyzed by EDS (Supplementary Figure S3). Elemental carbon, oxygen and magnesium were homogeneously distributed in PPGM electrospinning. Elemental magnesium appeared when the MgO NPs were added to the PLLA-Gelatin solution. Thus, the MgO NPs were incorporated within the electrospun fiber scaffolds observed in the TEM images of particles embedded within the fibers (Figure 2B) and microparticles attached to the fibers visible in the SEM images of the PPGM-L, PPGM-M and PPGM-H electrospun scaffolds.

Subsequently, the physicochemical properties of the PPGM fiber scaffolds were assessed. MgO NPs show a sharp diffraction peak at 2*θ* = 42.82° in the XRD pattern [28]. The peak observed in the fiber scaffolds doped with MgO NPs became more prominent as the amount of MgO NPs increased, which confirmed the incorporation of MgO (Figure 2C). The FTIR spectra of the MgO NPs and PPGM electrospun samples are shown in Figure 2D. The presence of gelatin in the fibrous membrane was indicated by the characteristic peaks of amide I at 1633 cm^−1^ and amide II at 1540 cm^−1^ [30]. The absorption peak at 3700 nm corresponded to the hydroxyl group of the hydrolysis product of MgO because MgO NPs are susceptible to absorbing water and hydrolyzing into magnesium hydroxide. The PPGM electrospun samples did not exhibit a clear, sharp peak because the MgO NP concentration did not reach 2% w/v [30]. These results agree with the SEM images, EDS analysis, and TEM images, confirming the successful preparation of PPGM electrospun fiber scaffolds.

The WCA test revealed the hydrophilic and hydrophobic properties of the PPGM fiber scaffolds (Figure 2E). The WCA of the monolayer electrospun PCL membrane exceeded 90°, indicating that the surface was hydrophobic. Water droplets applied to the PLLA-gelatin surface were rapidly absorbed upon superimposition of the PLLA-gelatin layer on the PCL electrospun membrane, demonstrating its excellent hydrophilicity. Meanwhile, there was little difference between the WCAs of the PPG and PPGM electrospun fiber scaffolds, indicating that adding MgO NPs did not affect the hydrophilicity of the PLLA-Gelatin membrane. Furthermore, the behavior of droplets falling on different surfaces of the PPGM membrane was also recorded by digital video (Supplementary Figure S4 and Supplementary Video S1). Droplets touching the hydrophilic surface diffused spontaneously and did not penetrate hydrophilic of the membrane. The droplet maintained its spherical shape on the hydrophobic surface, effectively blocking penetration in the opposite direction. This result demonstrated that the MgO NPs loaded within the hydrophilic membrane could traverse the hydrophobic layer and be transported downward into the trabecular bed [7]. Consequently, PPGM electrospinning relies on the outer PCL membrane to resist exogenous bacterial colonization and fluid infiltration. In contrast, the inner PLLA-Gelatin-MgO membrane absorbs chronic exudates from the wound area to a certain extent.

The thermal stability of the electrospun scaffolds was evaluated using thermogravimetry under extreme conditions (Figure 2F). The monolayer PCL membrane exhibited a higher initial degradation temperature than the other bilayer membranes. However, it showed lower stability and higher final mass loss. The final residual mass of the sample gradually increased with the incorporation of the inner PLLA-gelatin membrane and doping of MgO NPs, although the initial degradation temperature decreased with an increase in MgO NPs. The electrospun scaffolds of the PPGM-M group exhibited a low loss rate and good thermal stability based on the weight-loss derivative plot (Figure 2G).

The amount of Mg^2+^ released was used to calculate the scaffold degradation rat. The release of Mg^2+^ ions was determined by measuring the ion content in ultrapure water co-incubated with the scaffold for a fixed time (Figure 2H). This process mainly occurred during the early stages of incubation and was employed to mimic the infiltration of tissue fluid in the membrane during the nascent stages of wounding. In the PPGM-L group, Mg^2+^ ion release from the membranes peaked on day 1, whereas on days 2 and 3 in the PPGM-M and PPGM-H groups, respectively. This is consistent with the trend observed in the time course of the *in vitro* cell culture.

We prepared Janus electrospun membranes rapidly with the same working parameters and environment for the direct deposition of fibers. The formation of NP clusters during the spinning process disrupted the equilibrium between electrostatic forces and surface tension. The PCL membrane was stacked on top of the PGM membrane, with fibers interwoven between the two layers to create a mechanical tangle that increases the connection [31]. Concurrently, the moist environment created by the exudation of blood and tissue fluids from the wound infiltrates the membrane via osmosis. At the same time, the hydrogen bonds reinforce the bond between the inner and outer membranes [32].

### *In vitro* experiments

#### Excellent biocompatibility of PPGM electrospinning

Biocompatibility tests assess whether a material is potentially hazardous to living organisms. To verify cytocompatibility, this study cultured the PPG and PPGM electrospun nanofiber scaffolds with NIH/3T3s or HUVECs.

**Figure 3.**
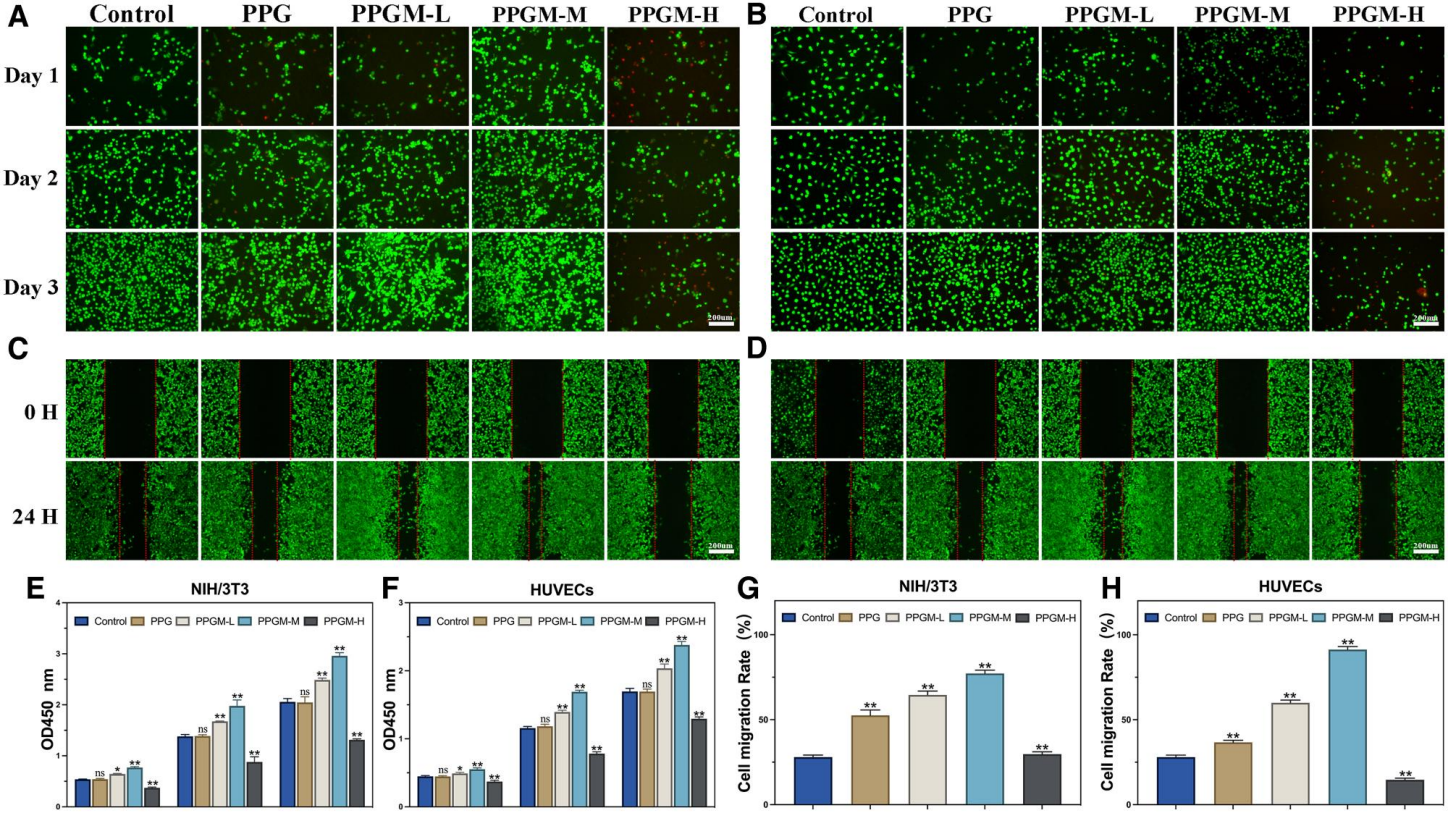
*In vitro* cell proliferation and migration. (**A**, **C**) Live/dead staining and cell migration images of NIH/3T3s. (**B**, **D**) Live/dead staining and cell migration images of HUVECs. (**E**, **F**) Cell Counting Kit-8 cytotoxicity test of NIH/3T3s or HUVECs on days 1, 3 and 5. (**G**, **H**) Cell migration rate at 24 h (*n* = 3, ns. represents no significance, *P <* *0.05, **P <* *0.01).

Live/dead staining performed on days 1, 2 and 3 confirmed PPGM electrospinning’s biocompatibility (Figure 3A and B). The PPGM-L and PPGM-M scaffolds exhibited high living cell (green) counts and a few dead cells (red). In contrast, the PPGM-H scaffold image had fewer viable and more dead cells. The OD values obtained from the CCK-8 assay revealed no significant differences between the Control and PPG groups (Figure 3E and F). However, the PPGM-L, PPGM-M and PPGM-H groups significantly differed from the control group on days 1, 3 and 5. The results of the CCK-8 and live/dead staining analyses were consistent with those of previous reports [33], and the behaviors above demonstrated that the PPGM-M electrospun scaffolds enhanced cell growth and proliferation. However, excessive concentrations led to an opposite outcome.

Subsequently, the cell migration capacity was analyzed via an established artificial wound (scratch assay) using NIH/3T3s or HUVEC monolayers. Figure 3C and D showed the NIH/3T3s and HUVECs scratch assay images in five groups after 0 and 24 h. The PPGM-L and PPGM-M scaffolds significantly enhanced cell migration in the scratch assay compared to the control group (Figure 3G and H). The migration of cells treated with the PPGM-H scaffold covered the wound area less than that of the Control group. Meanwhile, the blank material did not elicit negative cellular responses in the PPG group.

**Figure 4.**
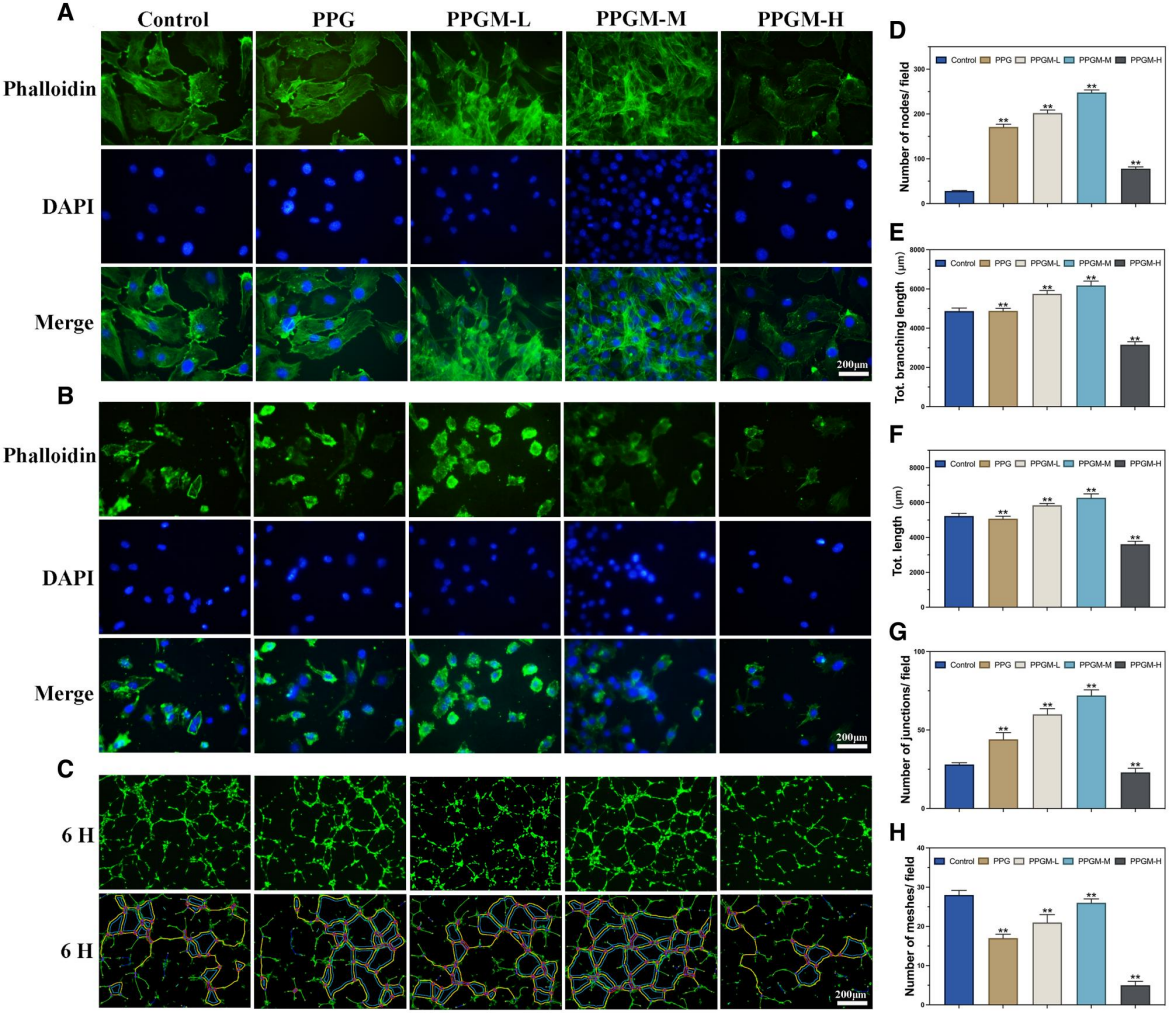
*In vitro* cell morphology and angiogenesis. (**A**) Cytoskeleton staining of NIH/3T3s. (B) Cytoskeleton staining of HUVECs. (**C**) Tube-forming and digital reconstruction of co-incubation HUVECs with different electrospinning at 6 h. (**D**–**H**) Statistical analysis of angiogenesis images at 6 h (*n* = 3, ns. represents no significance, **P <* *0.01).

Meanwhile, cell morphology was evaluated using actin filament staining (Figure 4A and B). The NIH/3T3 and HUVEC morphology and state did not change with the addition of PCL, PLLA, gelatin and MgO NPs, except for the PPGM-H group, owing to the accumulation of excess Mg^2+^. The cell stretching status was satisfactory, indicating excellent PPGM electrospun fiber scaffold biocompatibility with no significant variability.

#### Accelerated angiogenesis of PPGM electrospinning

The proangiogenic capacity of the PPGM electrospun fiber scaffold was evaluated through tube-forming experiments with HUVECs (Figure 4C). These results are consistent with those of the cell scratch experiments described in the previous section, indicating the ability of endothelial cells to migrate. Presentation in five directions based on the number of nodes/fields, total branching length, total length, number of junctions/fields, number of meshes/fields and the images of the PPG group after addition were not significantly different from those of the Control group (Figure 4D–H). This finding confirms that the material has high biocompatibility, consistent with previous experimental findings [34]. However, the values increased, peaked in the PPGM-M group and became negative in the PPGM-H group with the presence and elevation of magnesium ions released from the fiber scaffolds. In general, angiogenesis is the formation of new blood vessels from existing ones involved in migrating proliferated vascular endothelial cells from nearby normal tissues to the damaged area, where they differentiate [35]. The PPGM electrospun fiber scaffold was active in the cell proliferation and migration phases, promoting angiogenesis. The Mg ion concentrations released from each scaffold behaved as expected, with the PPGM-M group ultimately being non-biotoxic in conjunction with the release of the characterization section and the reported composite bone substitutes [36].

#### PPGM electrospinning mediated the macrophage phenotype towards M2

**Figure 5.**
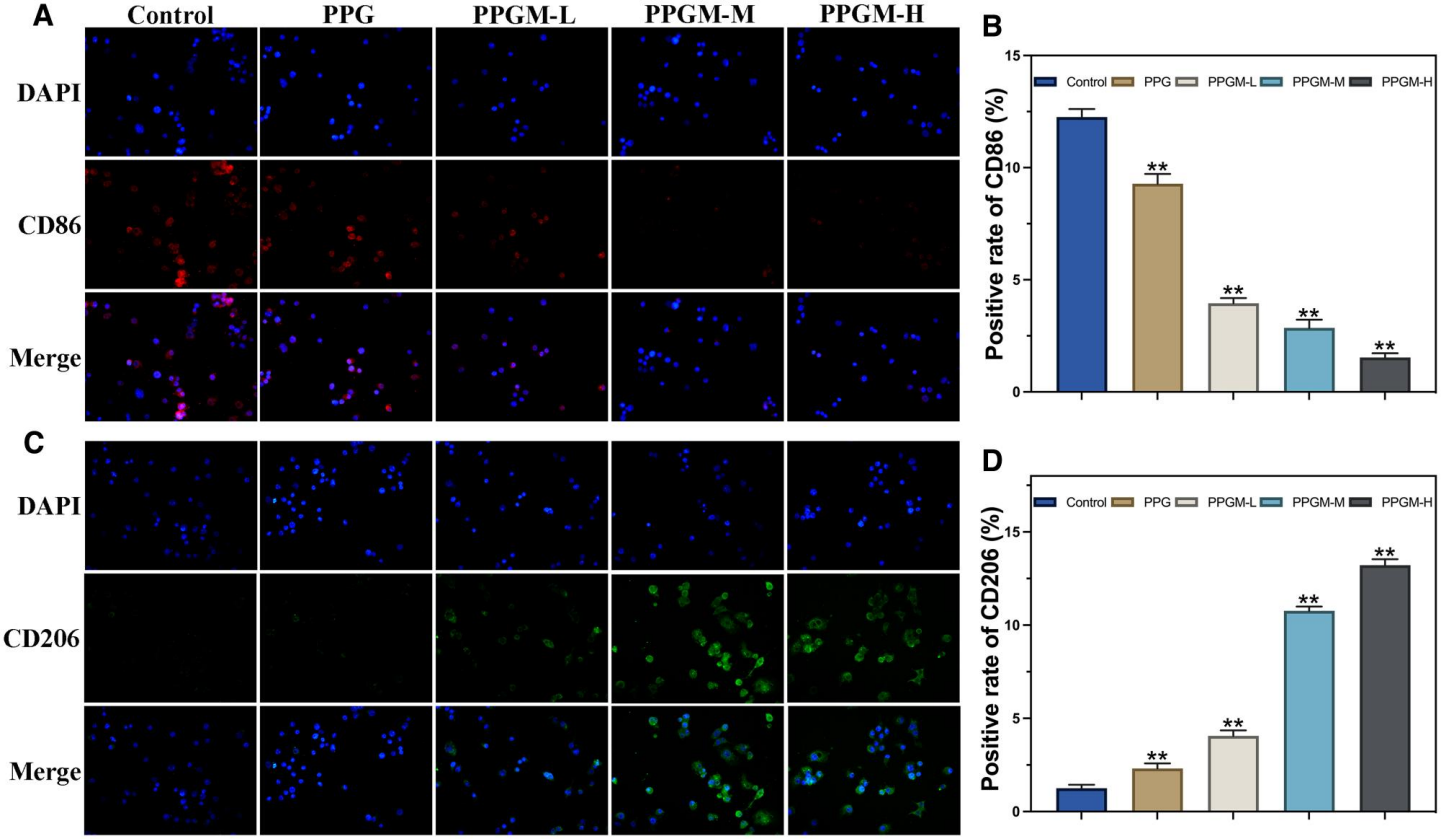
*In vitro* immunoregulatory mechanism of PPGM electrospinning. (**A**, **B**) M1 macrophage levels in RAW264.7 cells were detected via CD86 fluorescence intensity after exposure to electrospinning. (**C**, **D**) M2 macrophage levels in RAW264.7 cells detected by CD206 fluorescence intensity after exposure to electrospinning (*n* = 3, **P <* *0.01).

Achieving a controlled macrophage phenotype is essential to regulate the immune microenvironment. Fluorescence images of RAW264.7 macrophage polarization modulation were obtained to investigate the anti-inflammatory effect of PPGM electrospinning (Figure 5). Macrophages in the Control group underwent LPS-induced polarization from M0 to M1 with minimal M2 polarization. However, the fluorescence intensity of CD86 (M1 marker, green) sequentially decreased with the addition of electrospinning and MgO NPs, and the expression of CD86 gradually reduced as the MgO concentration increased (Figure 5A and B). The PPG scaffold contributed little to M2 polarization. In the presence of MgO NPs, there was a gradual increase in the proportion of M2-polarized cells and CD206 expression (M2 marker, red, Figure 5C). The MgO NPs from PPGM electrospinning caused the fluorescence intensity of CD206 to peak at a medium concentration of 0.5% w/v, indicating that the scaffold had the most vital ability to promote M2 polarization, which was consistent with previous cellular activity results (Figure 5C and D). Similarly, improving the inflammatory environment positively affected the formation of new blood vessels.

Although the electrospun scaffolds slowly released MgO NPs, loading large amounts of Mg^2+^ ions is detrimental to the cells. First, an inflammation-induced acidic environment will likely exacerbate inflammation progression [37]. The exposed MgO NPs quickly react with water to produce Mg^2+^ and OH^-^, creating a bias towards alkalinity to counteract the acidic environment [28]. This ultimately brings the wound microenvironment closer to the physiological state. Thus, the alkaline degradation products of MgO NPs could possibly cause a reduction in inflammation. Furthermore, macrophages are sensitive to ion concentration and particle size [38], as shown in studies of macrophage phenotype induction by magnesium, illustrating NF-kB p65 nuclear translocation [39]. This study showed no significant differences in the macrophage phenotypes in the Control and PPG groups, suggesting magnesium-induced immunomodulation. TRPM7 can be activated by Mg through the TRPM-PI3K-AKT pathway, thereby reducing the release of pro-inflammatory factors [40]. Immunomodulation by magnesium promotes the recruitment and activation of monocytes to macrophages. It reduces the release of numerous pro-inflammatory factors, whereas the presence of TRPM7 siRNA attenuates the immune effects of magnesium [41]. Therefore, macrophages may sense magnesium through TRPM7 and perform its functions.

#### Extensive bacterial killing by PPGM electrospinning

**Figure 6.**
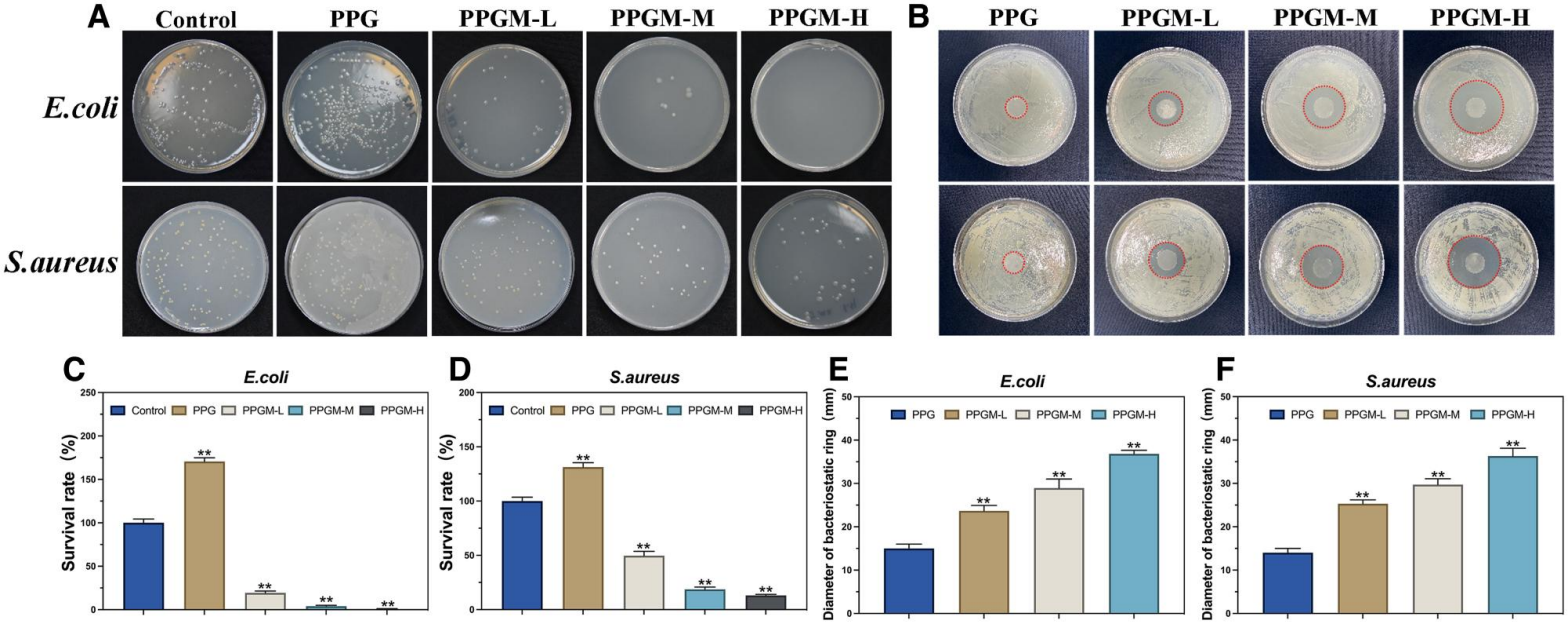
*In vitro* antimicrobial experiment of the PPGM electrospinning. (**A**) Photographs showing the colonies in the agar plate counting test for *E.coli* and *S.aureus*. (**B**) Photographs showing the colonies in the *E.coli* and *S.aureus* inhibition loop assay. (**C**, **D**) Survival rate of PPGM electrospinning against *E.coli* (C) and *S.aureus* (D). (**E**, **F**) Diameter of a bacteriostatic ring of PPGM electrospinning against *E.coli* (E) and against *S.aureus* (F) (*n* = 3, **P <* *0.01).

*Escherichia coli* and *S.aureus* are typical gram-negative and gram-positive organisms, respectively, and are common types of infecting organisms in clinical wound care. The PPGM scaffolds demonstrated antimicrobial activity against *E.coli* and *S.aureus* using agar plate counting and inhibition loop assays based on the number of surviving colonies and the radius of the circle of inhibition (Figure 6). The CFU number in the electrospun PPGM decreased as the loading of MgO NPs increased, significantly reducing the viability of the bacteria (Figure 6A, C and D). Notably, *E.coli* did not survive in the PPGM-H group, whereas *S.aureus* remained sporadically colonized. Similarly, the diameter of the circle of inhibition formed by the PPGM electrospun fiber scaffolds was larger for *E.coli* than for *S.aureus* (Figure 6B, E and F). However, no inhibitory ring formation was detected in the PPG-treated group (Figure 6B), indicating that PPG did not inhibit bacterial growth. This finding showed that the MgO NPs and Mg^2+^ in the PPGM electrospun scaffolds played a crucial role in the antibacterial properties and were superior against *S.aureus* than against *E.coli*.

The bactericidal capacity of the scaffolds depends on the concentration of Mg ions. The results of the antimicrobial experiments with PPGM-H differed from those of the cellular experiments. Unfortunately, we did not microscopically characterize the bacteria’s morphology on the fibrous membrane's surface. Nevertheless, based on the work of others [34], the SEM revealed a contracted bacterial morphology with an uneven surface. This could result from MgO NPs attaching to the bacterial cell membrane, which resulted in the death of bacteria due to a change in membrane permeability [42]. Moreover, MgO NPs demonstrated antimicrobial properties by catalyzing the formation of ROS [43]. In neutral or acidic environments, ROS catalyzed by MgO NPs formed hydroperoxyl radicals that react with peptide chains in the bacterial wall, leading to bacterial damage and death [44].

### *In vivo* experiments: PPGM electrospinning accelerated wound healing

**Figure 7.**
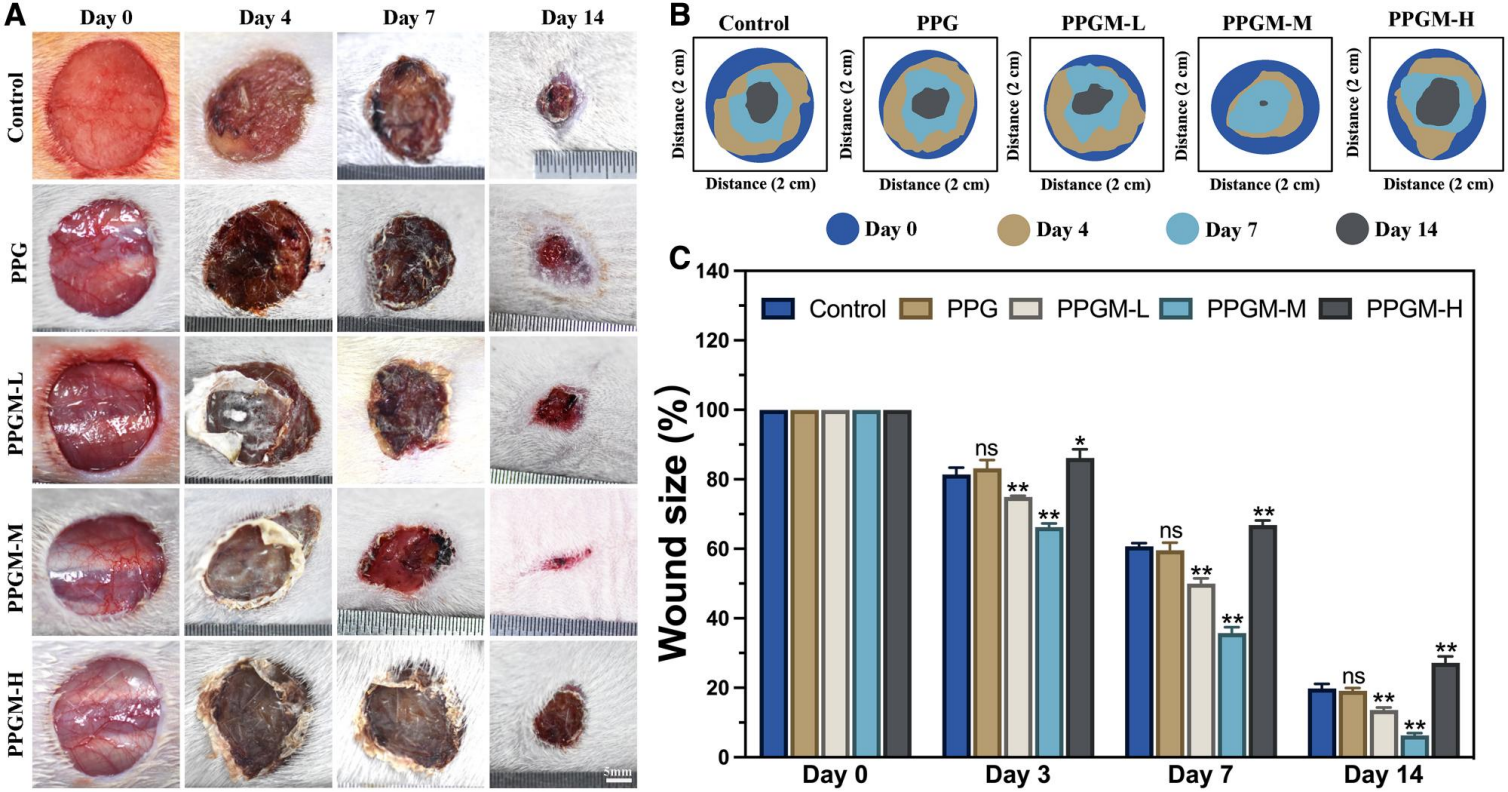
*In vivo* wound healing of PPGM electrospinning. (A) Corresponding representative photographs of Control, PPG, PPGM-L, PPGM-M and PPGM-H on days 0, 3, 7 and 14. (**B**) Wound area reconstruction schematic. (**C**) Wound size (*n* = 3, ns. represents no significance, **P <* *0.01).

Owing to electrospinning nanofibers display efficacious antibacterial and anti-inflammatory properties *in vitro*. Sprague Dawley rats were chosen as *in vivo* subjects to verify their wound-healing effect. Based on a handheld electrospinning device, the PPGM electrospun fiber membrane could be tailored to fit the needs of wounds of all shapes and sizes by varying the electrospinning deposition to cover the wound area (Supplementary Figure S5) completely. The wound healing rate in each group was observed macroscopically at various time points (Figure 7A), and reconstruction of the wound area was observed from digital images (Figure 7B). Over the 14-day healing period, there was a significant difference in the wound healing rate and efficacy of the PPGM electrospun fiber scaffolds as time progressed (Figure 7C). The wound-healing rate of the PPGM-M electrospun fiber scaffold was significantly higher than that of the other groups throughout the healing process. The PPGM-M group showed a significantly smaller wound area than the other groups and the Control group on day 3. On day 7, the wound area in the PPGM-M group remained less than 50%, and the wound had completely healed by day 14 after PPGM-M treatment but not in any of the other treatment or control groups. However, wound healing proceeded in the PPGM-H group at a slower rate owing to the excessive magnesium ion concentration, with a lower wound healing rate at all time points than that of the Control group (Figure 7C), and even demonstrated its toxic effect. The electrospun fiber scaffold remained stable for over 4 days, continuously releasing MgO NPs into the wound until the initial scab formed, according to the digital image of the wound.

In addition to regular circular wounds, we also established square, triangular, and polygonal curved wounds to validate the ability of PPGM-M electrospun membranes to promote irregular wound healing (Supplementary Figure S6). The wound area was adequately covered with PPGM membrane sprayed *in situ* using a handheld electrospinning device. The PPGM-M membrane significantly reduced the wound area compared to the Control group, ultimately accelerating the wound healing.

**Figure 8.**
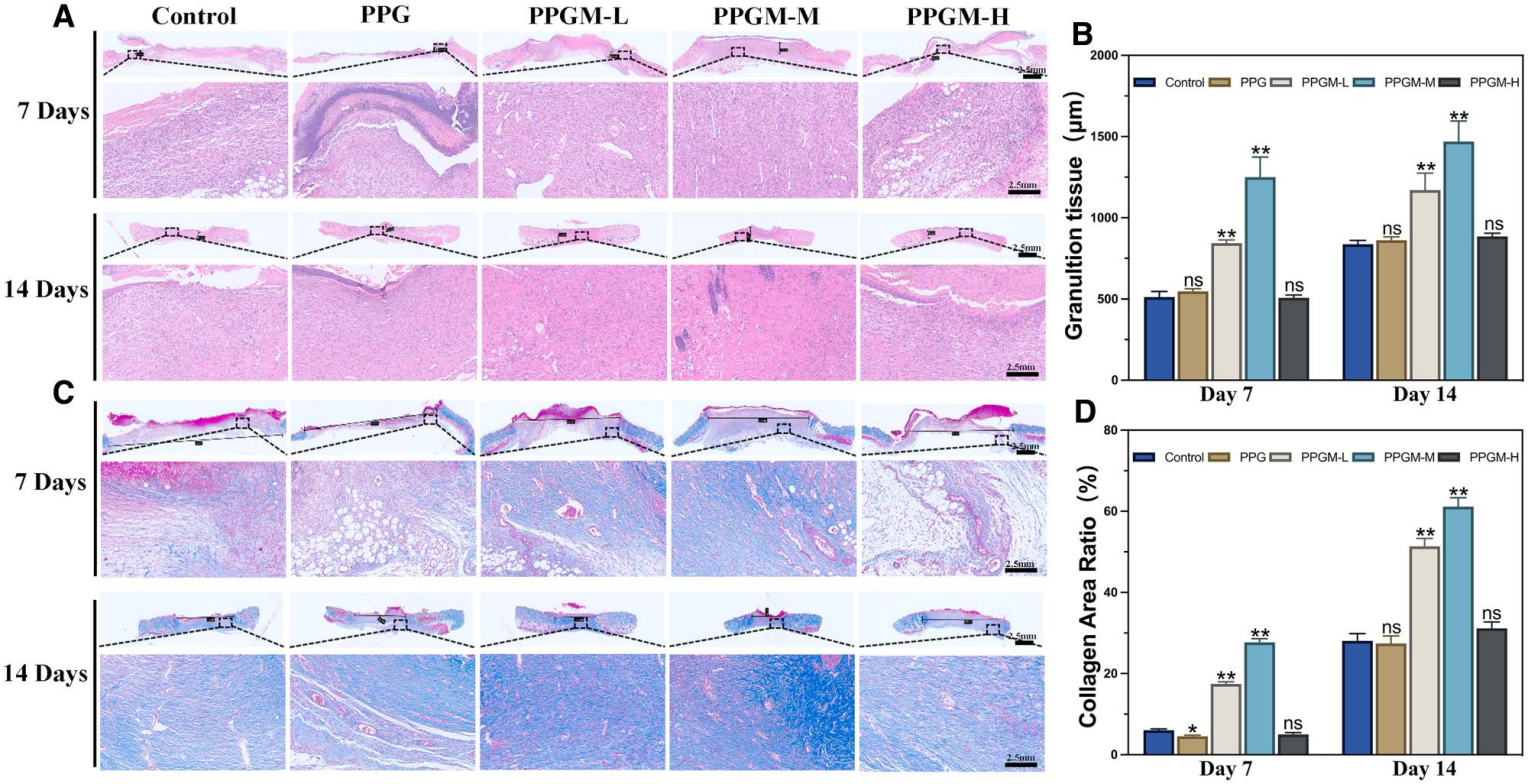
Histological analysis of wound by PPGM electrospinning on days 7 and 14. (**A**) Representative HE staining images. (**B**) Granulation tissue. (**C**) Representative Masson’s trichrome staining images. (**D**) Collagen area ratio (*n* = 3, ns. represents no significance, *P <* *0.05, **P <* *0.01).

Skin tissues were collected on days 7 and 14 after treatment. The skin structure was observed by HE staining (Figure 8A), and collagen deposition was detected by Masson’s trichrome staining (Figure 8B). The PPGM-M group had a well-organized and condensed skin structure that displayed elevated granulation tissue thickness according to HE staining (Figure 8C). Moreover, newly formed skin appendages were observed in the regenerated skin tissues on day 14. The other groups showed looser skin structure and a more significant proportion of immature tissue than the PPGM-M group; this suggested that PPGM-M electrospinning played an active role in the functional reconstruction of wounds. Masson’s trichrome staining revealed that the PPGM-M group exhibited a high density of neoplastic collagen fibers, which displayed an ordered wavy arrangement compared to the other groups. Additionally, the collagen area ratio in the PPGM-M group was significantly higher than that in the other experimental groups (Figure 8D).

**Figure 9.**
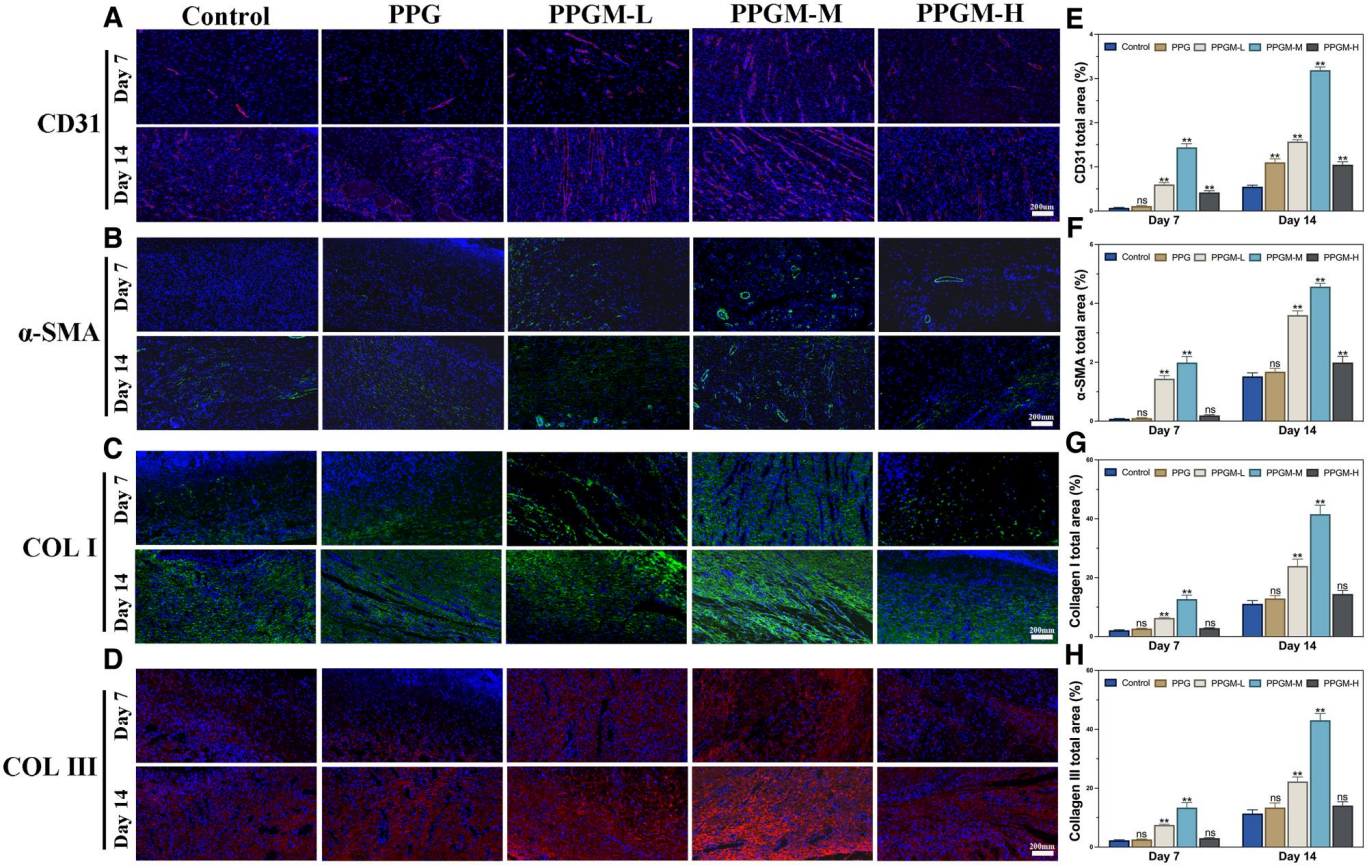
Immunofluorescence staining of neovascularization and collagen deposition under PPGM electrospinning. (**A**) Representative CD31 immunofluorescence images and E) their semiquantitative statistics in the total area. (**B**) Representative α-SMA immunofluorescence images and (**F**) their semiquantitative statistics in the total area. (**C**) Representative COL I immunofluorescence images and (**G**) their semiquantitative statistics in the total area. (**D**) Representative COL III immunofluorescence images and (**H**) their semiquantitative statistics in the total area (*n* = 3, ns. represents no significance, **P <* *0.01).

Prompt establishment of a vascular network is essential for the early transport of nutrients and waste to the wound site, which is the limiting step for regenerative applications [45]. Magnesium ions have been shown to have a role in promoting vascular regeneration and remodeling of the extracellular matrix during the proliferative period [46, 47]. CD31 is a marker of vascular endothelial cells; immunofluorescence staining further verified its level to reflect angiogenic progression during wound healing. Meanwhile, the degree of vascularization can also be reflected by α-SMA, a marker of vascular smooth muscle cells. The red fluorescence of CD31 in the PPGM-M group was significant on days 7 and 14 compared to that in the other groups (Figure 9A and E), indicating a higher percentage of neovascularization in terms of number and area. The α-SMA expression in five groups was consistent with that of CD31 (Figure 9B and F). α-SMA produced by smooth muscle cells matures later than endothelial cells; therefore, its expression intensity was weaker than CD31 at the same time point. Next, collagen types I and III are crucial dermis constituents, forming the extracellular matrix’s primary structural proteins. Collagen I (green, Figure 9C) and collagen III (red, Figure 9D) tended to be expressed more over time. The arrangement of the collagen fibers was pike-shaped and wavy, indicating collagen deposition and remodeling. This was particularly evident for the PPGM-M group, which performed best. Elevated collagen type III levels during the initial phases of wound healing can help minimize subsequent scarring [48]. Collagen fluorescence-stained images with gross wound images and HE staining showed that a large amount of nascent collagen contributed to the remodeling of the extracellular matrix, reducing scarring and wound size. Therefore, the PPGM-M electrospun fiber scaffold accelerated angiogenesis and promoted collagen deposition.

**Figure 10.**
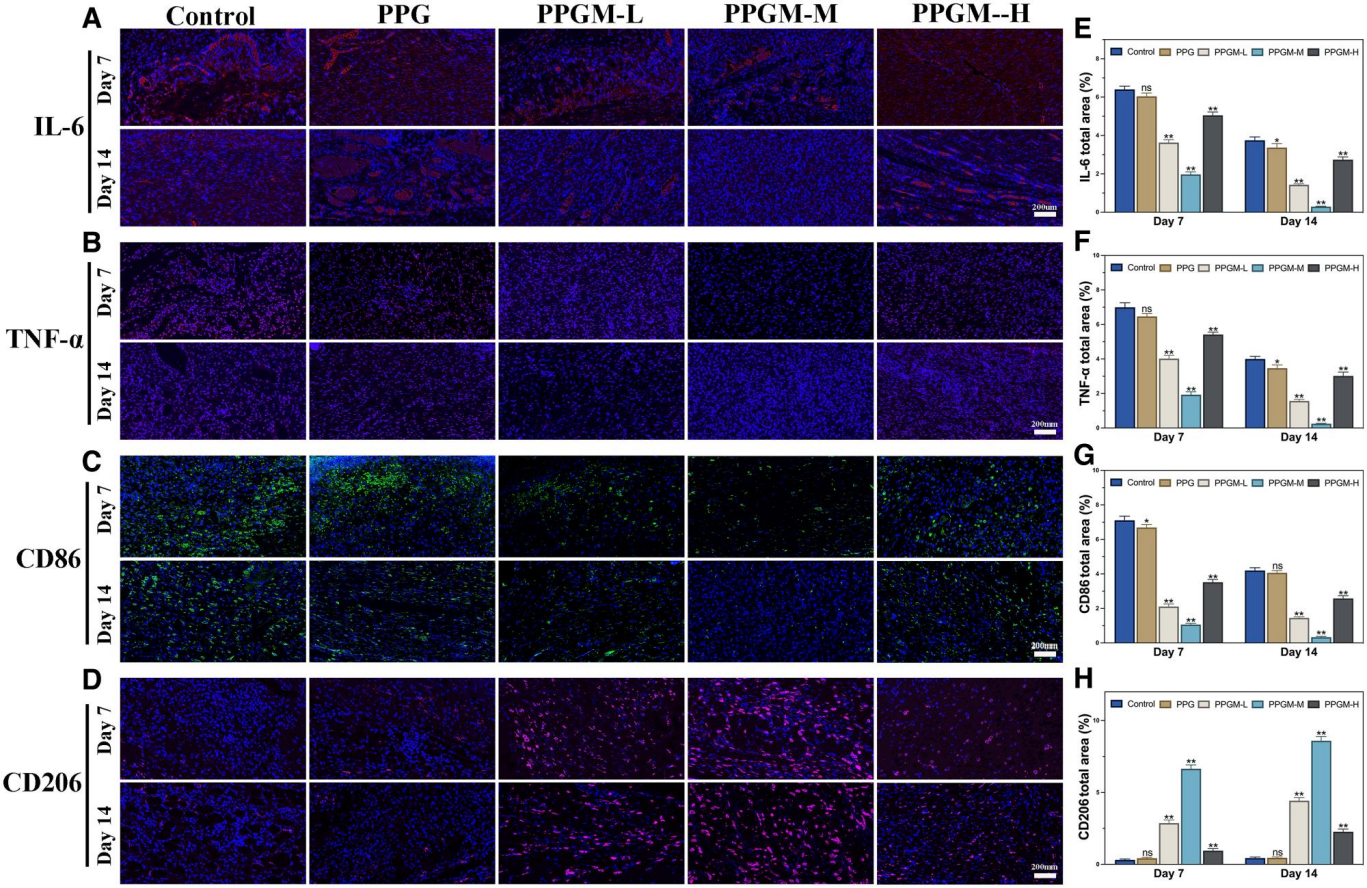
Immunofluorescence staining showing immunomodulation by PPGM electrospinning. (**A**) Representative IL-6 immunofluorescence images and (**E**) their semiquantitative statistics in the total area. (B) Representative TNF-α immunofluorescence images, and (**F**) their semiquantitative statistics in the total area. (**C**) Representative CD86 immunofluorescence images and (**G**) their semiquantitative statistics in the total area. (**D**) Representative CD206 immunofluorescence images and (**H**) their semiquantitative statistics in the total area (*n* = 3, ns. represents no significance, *P <* *0.05, **P <* *0.01).

Environments with high levels of inflammation adversely affect wound healing. To further investigate the level of inflammation in different treated wounds, the expression of inflammatory factors interleukin 6 (IL-6) and tumor necrosis factor-α (TNF-α) decreased over time in each group, with a significant decrease observed in the PPGM-M group (Figure 10). Green fluorescent CD86, IL-6 and TNF-α exhibited a decreasing trend, while red fluorescent CD206 showed a gradual increase in expression as the scaffolds changed from PPG to PPGM-M. These results confirmed that the PPGM-M group showed excellent efficacy in reducing the production of pro-inflammatory factors and promoting macrophage transformation from M1 to M2, indicating superior anti-inflammatory efficacy compared to the other groups.

Wound recovery is facilitated by a favorable immune microenvironment and abundant neovascularization, which can be attributed to the regulatory role of Mg^2+^. The PPGM electrospun scaffold provided nutrients to the granulation tissue by facilitating the formation of new blood vessels; this accelerated cell migration and reduced the length of the visible wound. Meanwhile, the scaffold reversed dysfunctional macrophage phenotypic switching by modulating the immune microenvironment. This facilitated macrophage conversion to the M2 phenotype and provided a solid foundation for later phases. Therefore, the application of PPGM electrospun fiber scaffolds during wound repair promoted the formation of new blood vessels and collagen remodeling and regulated the immune microenvironment, thereby enhancing vascularization and epithelialization to promote wound healing.

## Conclusions

A handheld electrospinning device was used to deposit MgO-doped fibrous membranes precisely onto acute wounds for wound management. Electrospun membranes have an extracellular matrix-like nanofibrous structure and play various roles depending on the constituent materials. Among them, the hydrophobic PCL fiber membrane prevents foreign contamination while promoting unidirectional drug transport in the inner layer. The incorporation of the inner hydrophilic membrane along with MgO NPs did not alter the physicochemical properties of the fibrous membranes. It improved the inherent antimicrobial capacity of the material and the bioactivity of the skin constituent cells *in vitro*. The electrospun PPGM scaffold significantly reduced inflammation and accelerated vascularization and epithelialization by altering the direction of macrophage polarization *in vivo*. Therefore, the current study showcases the potential of PPGM electrospun scaffolds for therapeutic applications in treating acute irregular wounds and personalized healthcare.

## Supplementary Material

rbae107_Supplementary_Data
