# Supplementary material for: In situ MgO nanoparticle-doped Janus electrospun dressing against bacterial invasion and immune imbalance for irregular wound healing
Source: Regen Biomater. 2024 Aug 23;11:rbae107. doi: 10.1093/rb/rbae107 (PMC11379472; doi:10.1093/rb/rbae107)
Supplement: rbae107_Supplementary_Data [file rbae107_supplementary_data.zip › Supplementary Material.docx]

**Supplementary Material:**

**In situ MgO** **nanoparticle-doped Janus electrospun dressing against bacterial invasion and immune imbalance for irregular wound healing**

Tao Zhou^1,3,^**^†^**, Yedan Chen^1,2,^**^†^**, Liangmin Fu^1,3,^**^†^**, Shan Wang^2,4^, Haihu Ding^3^, Qiaosheng Bai^3^, Jingjing Guan^1,3,^*, Yingji Mao^1,2,4,^*

^1^ Department of Orthopedics and Department of Plastic Surgery, The First Affiliated Hospital of Bengbu Medical University, Bengbu, China

^2^ School of Life Sciences, Bengbu Medical University, Bengbu, China

^3^ Anhui Province Key Laboratory of Tissue Transplantation, Bengbu Medical University, Bengbu, China

^4^ Anhui Nerve Regeneration Technology and Medical New Materials Engineering Research Center, Bengbu Medical University, Bengbu, China

**^†^** Tao Zhou, Yedan Chen and Liangmin Fu contributed equally to this work.

***Corresponding Authors:**

Jingjing Guan, Department of Orthopedics and Plastic Surgery, The First Affiliated Hospital of Bengbu Medical University, Bengbu, Anhui 233004, China; Tel: +86-552- 3175396; Email: Xiaogui1983.cool@163.com;

Yingji Mao, School of Life Sciences, Bengbu Medical University, Bengbu, Anhui 233030, China; Tel: +86-552-3175396; Email: myj123@bbmc.edu.cn.

**
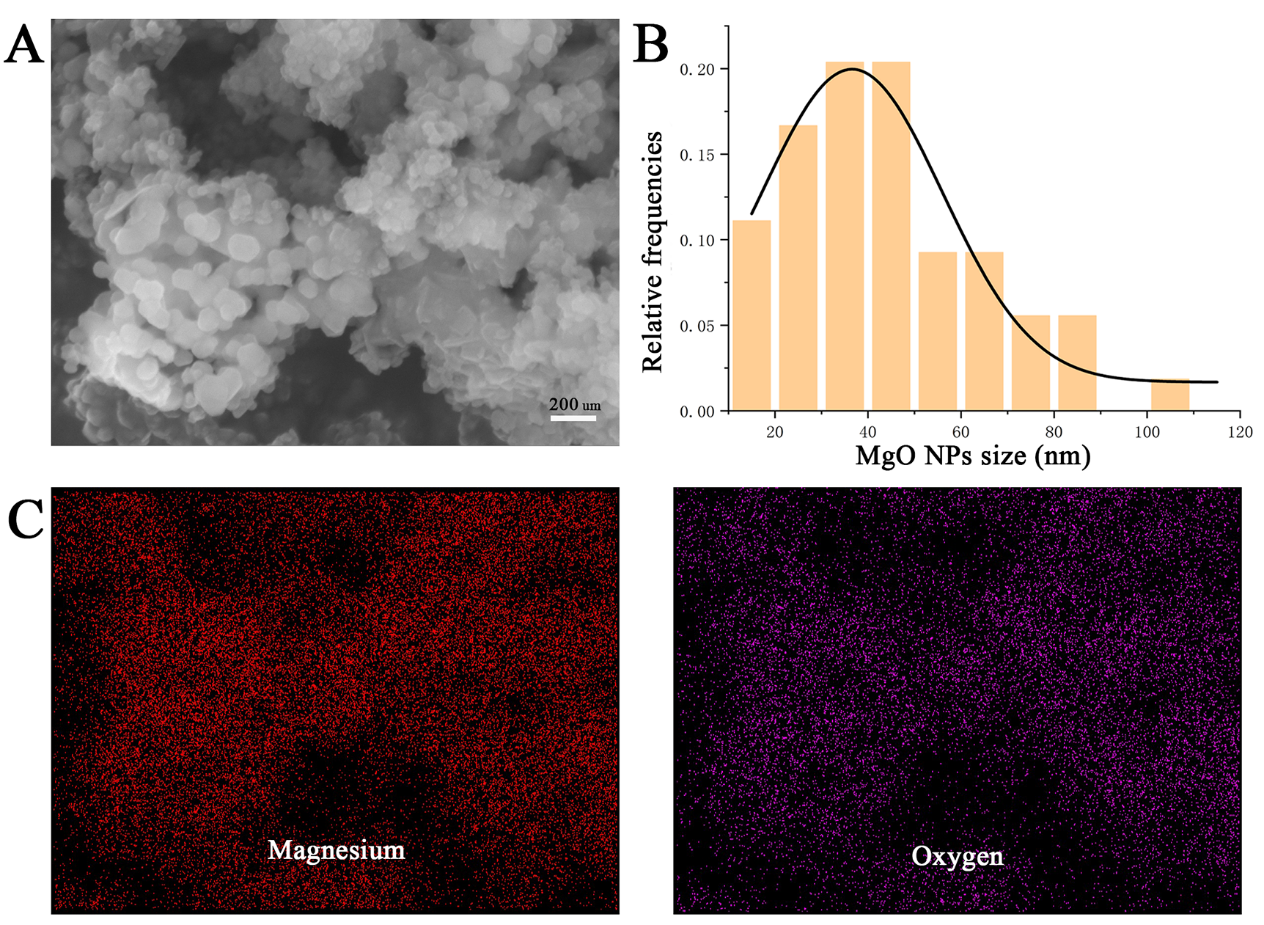
**

**Figure S1.** MgO NPs characterization. A) Representative SEM image. B) Nanoparticle size distribution. C) EDS elemental mapping of Mg and O.


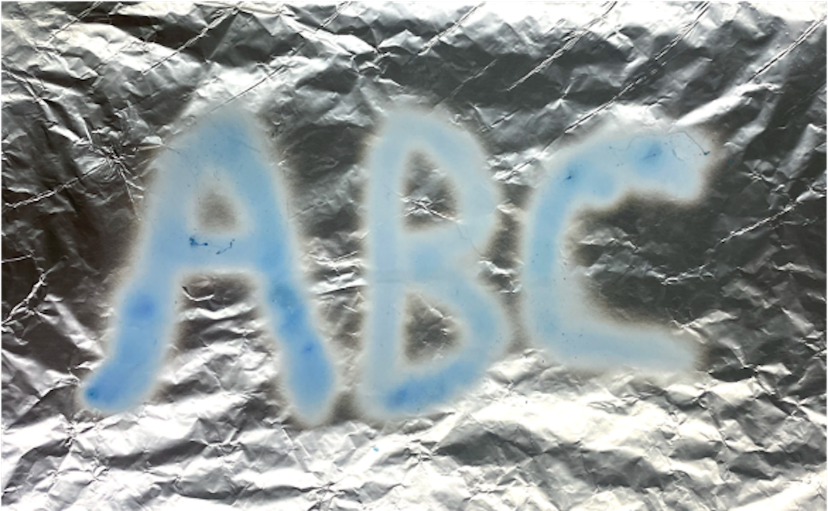


**Figure. S2.** The example of customizable in situ electrospinning.

**
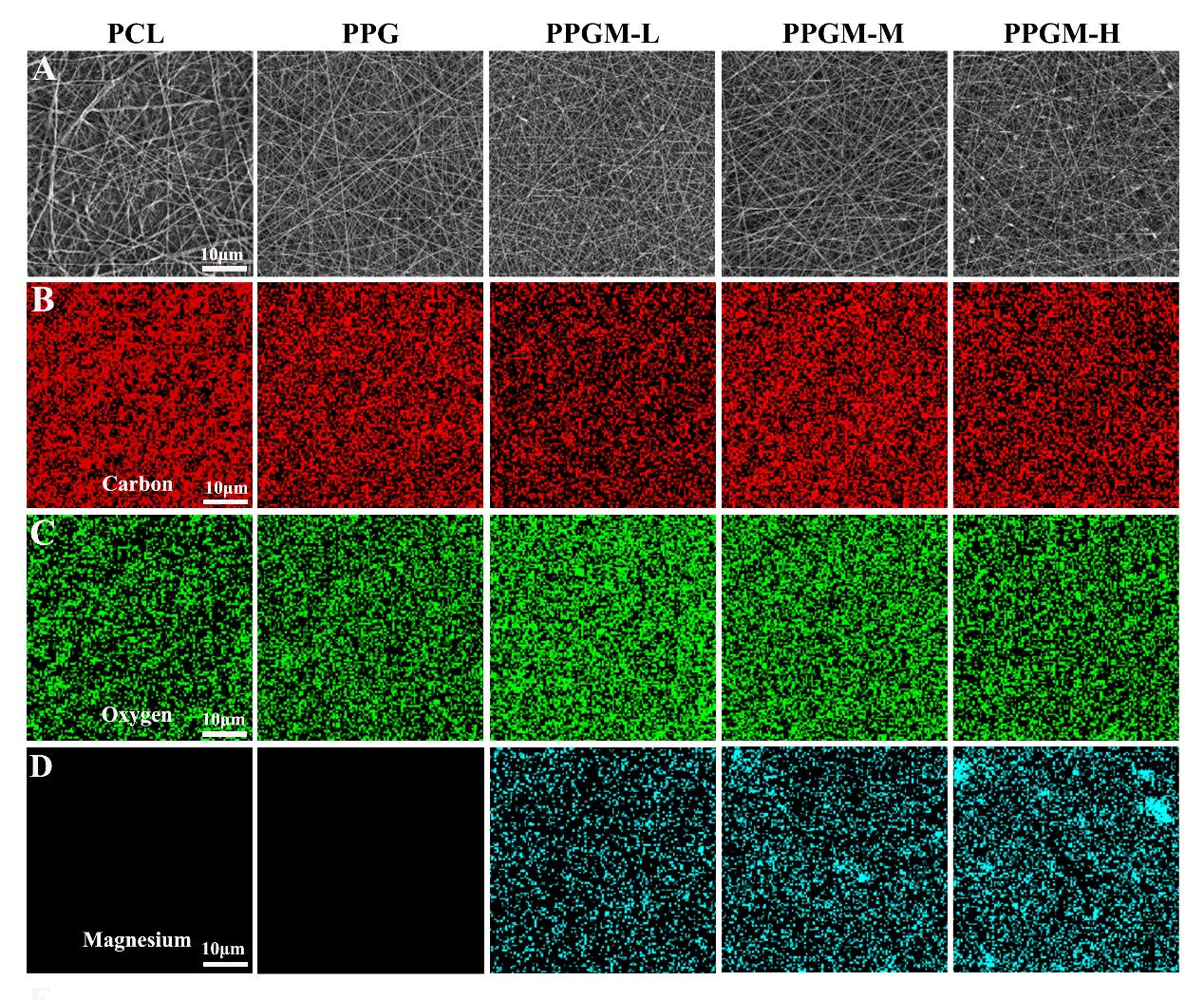
**

**Figure S3.** Morphology of PPGM electrospinning. A) Representative SEM images. B-D) Energy-dispersive X-ray spectroscopy elemental mapping of C, O, and Mg.


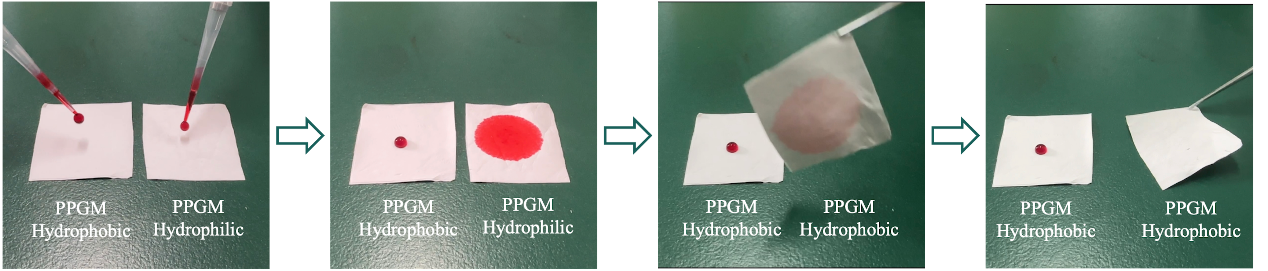


**Figure S4.** Representative video capture images show that the droplets cannot be transported from the hydrophilic to the hydrophobic surface of the PPGM membrane.

**
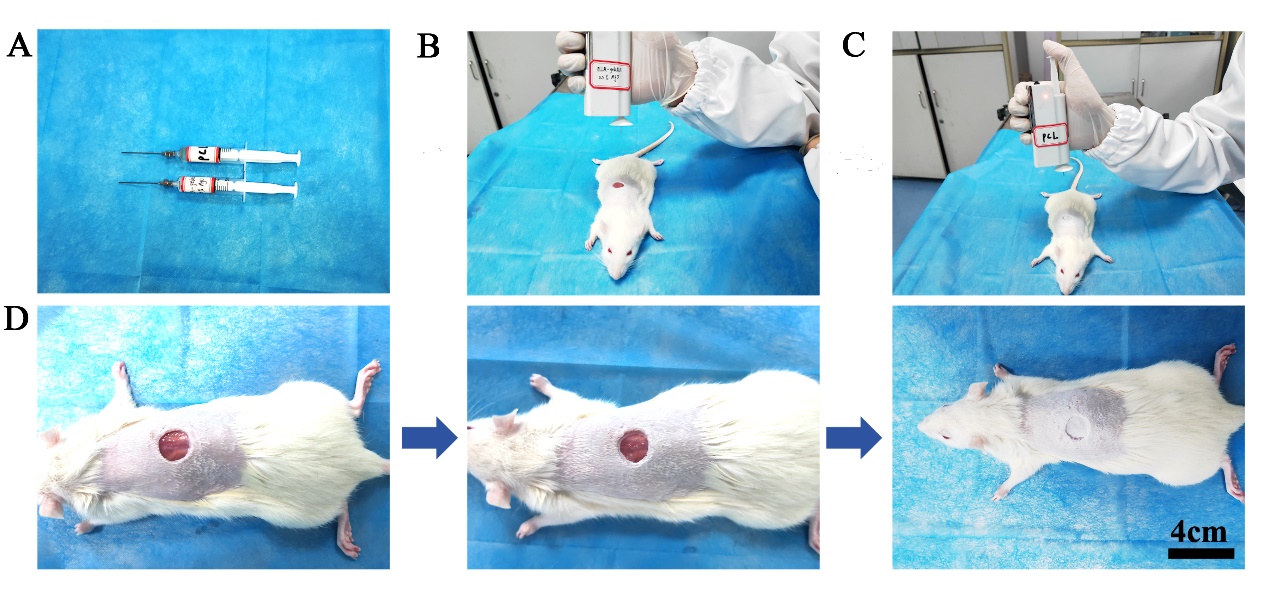
**

**Figure. S5.** Rat modeling flowchart. A) Homogenized PCL solution and PLLA-Gelatin-MgO solution in 5 mL syringes. Real-time images of hydrophilic PLLA-Gelatin-MgO electrospun membrane B) and hydrophobic PCL electrospun membrane C) spraying with a handheld electrospinning device on a dorsal skin defect. D) Images of the in situ PPGM electrospun scaffold preparation.


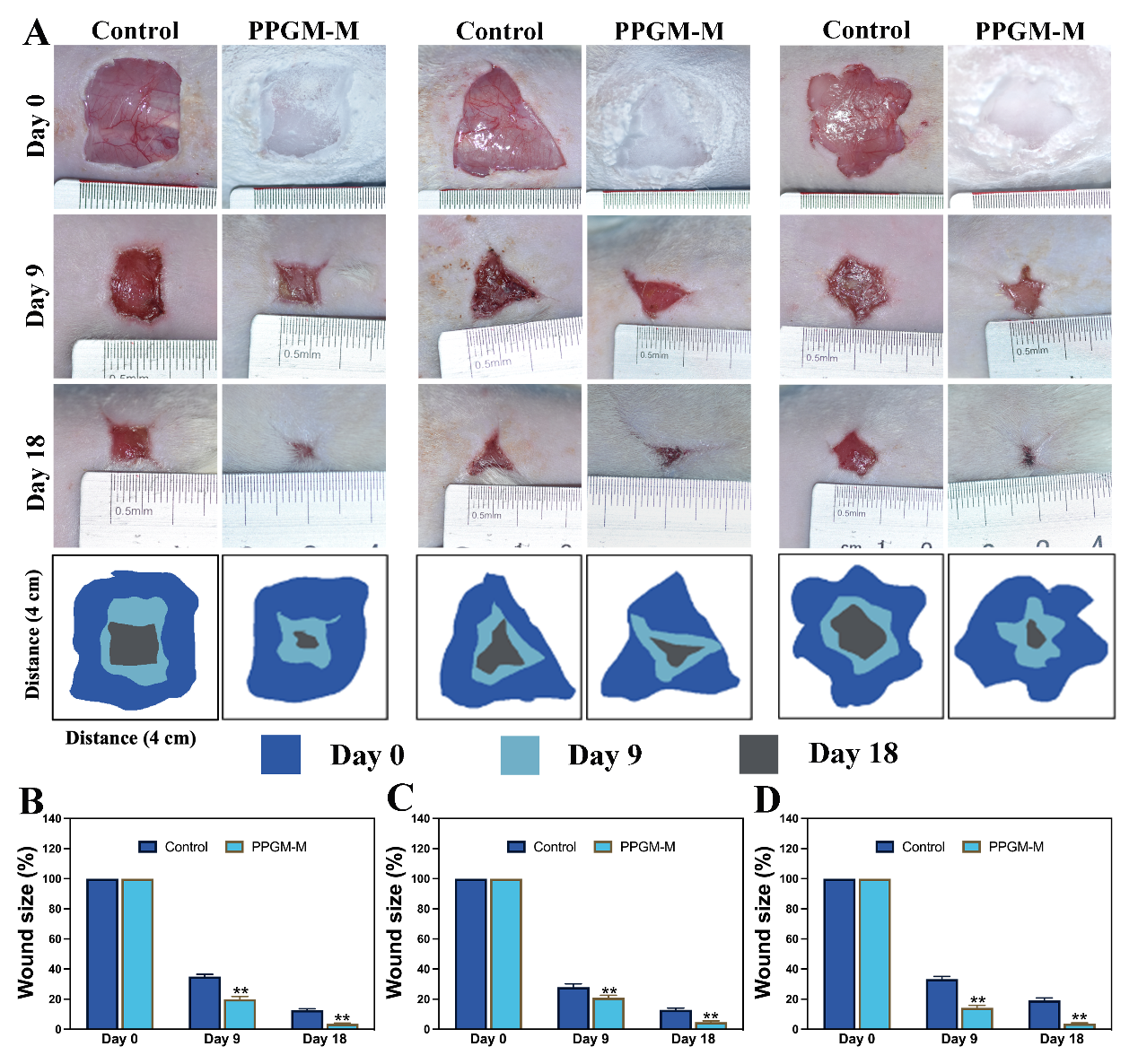


**Figure S6.** In vivo irregular wound healing of PPGM-M electrospinning. A) Corresponding representative photographs and reconstruction schematic of Control and PPGM-M. B) Statistical diagram of the square wound area. C) Statistical diagram of the triangular wound area. D) Statistical diagram of the polygonal curved wound area. (n = 3, ***P* < 0.01)
